# Supplementary material for: Identification of Freezing-Responsive microRNAs and Their Targets in Chinese Jujube by Small RNA and Degradome Sequencing
Source: Int J Mol Sci. 2026 Sep 4;27(17):7912. doi: 10.3390/ijms27177912 (PMC13566890; doi:10.3390/ijms27177912)
Supplement: Supplementary file 1 [file ijms-27-07912-s001.zip › Supplementary Material S2/novel_miR_201.pdf]

Provisional ID	:	novel_miR_201
Score total	:	0.3
Score for star read(s)	:	-1.3
Score for read counts	:	0
Score for mfe	:	0
Score for randfold	:	1.6
Score for cons. seed	:	
Total read count	:	100
Mature read count	:	100
Loop read count	:	0
Star read count	:	0

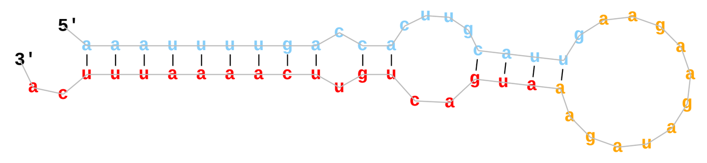

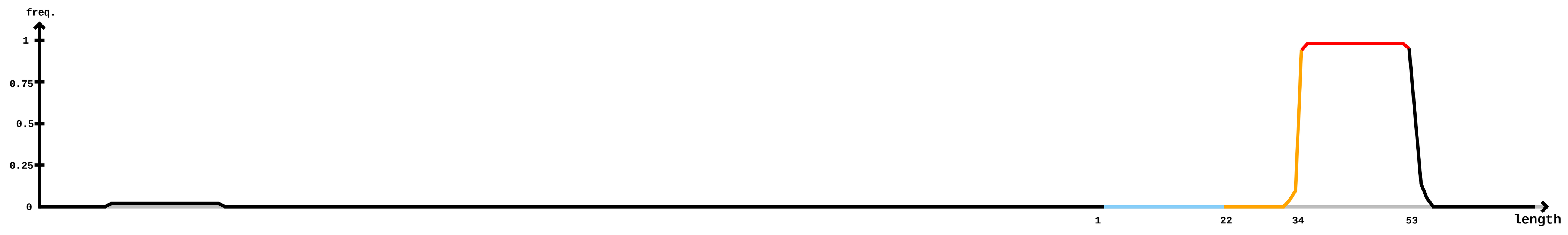

Star Mature

5'- cccaaaaaaacaaaaaaaaaaaaaaauauagaaaaucaccauaaaaauugauugaaacccagauacgcuaacaagaugcaacauaucaauuuuaaaccuacaaaaauuagccuauaaauuaauuaauauaaguugcaaauuacaagaauauugauuugcaaugaacuacauacggagaaaaauuuugaccacuugcauugaagaagauagaaaugacuguucaaaauuucaccuguaucuuugagguuuuuca -3' exp

	reads	mm	sample
..........caaaaaaaaaaaaaauGuag.......	2	1	LCK-3
.......aaaugacuguucaaaauuuc.......	1	0	JCK-1
.......aaaugacuguucaaaauuuc.......	1	0	JCK-2
.......aaaugacuguucaaaauuucac.......	1	0	LT-3
.......aaaugacuguucaaaauuucacc.......	1	0	LT-1
........aaugacuguucaaaauuuc.......	1	0	LT-2
........aaugacuguucaaaauuuca.......	3	0	JT-3
........aaugacuguucaaaauuucac.......	1	0	LT-1
........aaugacuguucaaaauuucacc.......	1	0	LT-1
.........augacuguucaaaauuuca.......	42	0	LCK-1
.........augacuguucaaaauuuca.......	1	0	LT-3
.........augacuguucaaaaNuuca.......	2	1	LCK-1
.........augacuguucaaaNuuuca.......	1	1	LCK-1
.........augacuguucaaaauuuca.......	31	0	LCK-2
.........augacuguucaaaauuucac.......	1	0	LT-3
.........augacuguucaaaauuucacc.......	1	0	LT-3
.........augacuguucaaaauuucacc.......	5	0	LT-1
.........augacuguucaaaauuucaccu.......	1	0	LT-1
.........augacuguucaaaauuucaccu.......	1	0	LT-3
..........ugacuguucaaaauuucacc.......	1	0	LT-3
..........ugacuguucaaaauuucaccu.......	3	0	LT-1
